# Mitochondrial quality control in stroke: From the mechanisms to therapeutic potentials

**DOI:** 10.1111/jcmm.17189

**Published:** 2022-01-17

**Authors:** Heyan Tian, Xiangyu Chen, Jun Liao, Tong Yang, Shaowu Cheng, Zhigang Mei, Jinwen Ge

**Affiliations:** ^1^ 118393 Key Laboratory of Hunan Province for Integrated Traditional Chinese and Western Medicine on Prevention and Treatment of Cardio‐cerebral Disease Hunan University of Chinese Medicine Changsha China

**Keywords:** mitochondrial biogenesis, mitochondrial dynamics, mitochondrial quality control, mitophagy, stroke

## Abstract

Mitochondrial damage is a critical contributor to stroke‐induced injury, and mitochondrial quality control (MQC) is the cornerstone of restoring mitochondrial homeostasis and plays an indispensable role in alleviating pathological process of stroke. Mitochondria quality control promotes neuronal survival via various adaptive responses for preserving mitochondria structure, morphology, quantity and function. The processes of mitochondrial fission and fusion allow for damaged mitochondria to be segregated and facilitate the equilibration of mitochondrial components such as DNA, proteins and metabolites. The process of mitophagy is responsible for the degradation and recycling of damaged mitochondria. This review aims to offer a synopsis of the molecular mechanisms involved in MQC for recapitulating our current understanding of the complex role that MQC plays in the progression of stroke. Speculating on the prospect that targeted manipulation of MQC mechanisms may be exploited for the rationale design of novel therapeutic interventions in the ischaemic stroke and haemorrhagic stroke. In the review, we highlight the potential of MQC as therapeutic targets for stroke treatment and provide valuable insights for clinical strategies.

## 1 INTRODUCTION

Stroke, a leading culprit of disability and mortality in China, causes 44 million physical disabilities and 5.5 million deaths around the world per year,^1^ which is a disease of immense public health significance with serious social and economic consequences.^2^ A Nationwide Population‐Based Survey of Stroke in China (NESS‐China) involved 480,687 adults in stroke from 2012 to 2013, has conveyed that the age‐standardized prevalence, mortality and incidence rates were 1114.8 cases per 100,000,114.8 cases per 100,000 and 246.8 cases per year respectively.^3^ This manufacture is a heavy burden on low‐ and middle‐income family in society. In this sense, establishing rational understanding on strokes can effectively prolong human life expectancy and boost economic development in the whole world.

Strokes can be broadly classified as ischaemic or haemorrhagic,^4^ accounting for about 80% and 20% of total strokes respectively.^5^ Stroke is usually caused by the focal brain dysfunction due to the death of cells triggered by the blockage or rupture of cerebral blood vessels. The interruption of blood flow or cerebral haemorrhage disturbs cellular homeostasis, and thus causes the pathophysiological processes, including oxidative stress, inflammation, excitotoxicity, apoptosis and other types of cell death (which may be necrotic, autophagic or associated with mitosis).^6^ Tissue‐type plasminogen activator (tPA) is the only thrombolytic drug which is approved for clinical treatment of ischaemic stroke. However, owing to the narrow therapeutic window (<4.5 h) and haemorrhagic transformation, its usage has been restricted in the treatment of ischaemic stroke.^7^ Haemorrhagic stroke has established far less devotion than ischaemic stroke. By any possibility cerebral haemorrhage, surgical decompression is still the current preferred treatment option.^8^ However, early resection of hematoma and local decompression as an early treatment method for haemorrhagic stroke cannot significantly improve the long‐term prognosis. Brain injury following haemorrhagic stroke and surgery is a changeable procedure involving a cascade of multipart pathological pathways and biochemical and metabolic measures, which is approximately categorized as decrease of blood flow, free radical injury, haemorrhagic neuroinflammation, neuronal apoptosis and brain herniation.^9^ Hence, it is critical to find a new target underlying the understanding new machinery for the treatment of stroke.

Mitochondria, as the powerhouse of the active cell, play a vital part in pathological conditions once stroke triggered in human body. During stroke, the internal balance system of cell is disrupted owing to the reduced supply of blood and the disturbed synthesis of adenosine triphosphate (ATP) in mitochondrial. Besides, the mitochondrial can regulate the cell death mechanism, including apoptosis and autophagy. Owing to preserving mitochondrial function is very important for neurological promotion and cell survival after stroke. In this sense, controlling the mitochondrial state during/after stroke can be a promising therapeutic strategy to stroke.

Therefore, mitochondria have been regarded as imperative goals for the advancement of new therapeutic interventions for stroke. Recent researches have suggested that mitochondrial quality control (MQC) is the origin for sustaining the steadiness and integrity of mitochondrial function and structure, and is a vital security machinery for cells to survive from mitochondrial damage.^10^ In this review, we will concentrate on the part of mitochondria in cell survival and cell death after stroke, and highlight the enhancement of mitochondria‐based in stroke.

## 2 MITOCHONDRIAL QUALITY CONTROL AND STROKE

### 2.1 Mitochondrial quality control systems

Mitochondrial quality control, including mitochondrial fission and fusion, mitochondrial biogenesis, and mitochondrial autophagy (mitophagy), is considered as the keystone on maintaining the integrity and stability of morphology, quantity and function, and plays an essential role in resistance mechanism for cells to survive from mitochondrial damage.^11^

The function of peroxisome proliferator‐activated receptor γ coactivator‐1α (PGC‐1α), the chief regulator of mitochondrial biogenesis, is activated via different proteins such as silent information regulator 2 homolog 1 (SIRT1), AMP‐activated protein kinase (AMPK), nuclear respiratory factor 1/2 (NRF‐1/2) and the mammalian target of rapamycin complex 1 (mTORC1) to maintain appropriate cellular homeostasis. PGC‐1α allows mitochondrial biogenesis to reach co‐regulation between nuclear activation and mitochondria via the PGC‐1α‐NRF‐1/2‐mitochondrial transcription factor A (TFAM) pathway.^12^ SIRT1 is an upstream regulator of PGC‐1α. When injury or proteostatic stress occurs, SIRT1 is overexpressed, enhancing the deacetylation of PGC‐1α and promoting mitochondrial biogenesis.^13^ AMPK is considered another chief regulator of mitochondrial biogenesis. Mitochondria biogenesis is enthused by AMPK to rise cellular energy production.^14^ Meanwhile, it is increased through upregulating the mTORC1/PGC‐1 signalling pathway.^15^

Mitochondrial fusion/fission is the basis of mitochondria dynamics, which controls mitochondrial networks and the cellular bioenergetics via the actions of dynamin‐related protein 1 (Drp1), fission 1 (Fis1), mitochondrial fission factor (Mff), mitofusin 1 and 2 (Mfn1/2) and optic atrophy 1 (OPA1).^16^ Mitochondria are dynamic organelles that require the balance of fission and fusion to adapt to proper function, cell growth, division and injury response. Drp1 is recruited to the outer mitochondria membrane (OMM) and formats a complex via an interaction with the OMM receptors Mff and Fis1. Hydrolysis of the Drp1‐bound guanosine triphosphate (GTP) contracts the Drp1 and permits severing of the enclosed membranes leading to mitochondrial fission. Mfn1 and Mfn2 proteins, that include two transmembrane domains in the OMM with a GTPase domain and are oriented to the cytoplasm, allow energy for the OMM fusion via performing with mitochondria lipid bilayer mixing. Similarly, OPA1 performs a similar action to allow inner mitochondria membrane (IMM) fusion.

Mitophagy can selectively remove the dysfunctional and damaged mitochondria and require the coordinated action between several mitophagy pathways activation including PTEN‐induced kinase 1 (PINK1)/Parkin, (BNIP3)/NIX and FUNDC1.^17^ Mitochondrial membrane potential (DΨm) is changed after mitochondrial injury, causing the activation of pink1. The target proteins of pink1 (Ubiquitin/Ub and Mfn2) recruit E3 ligase parkin to the mitochondrial outer membrane. Parkin can ubiquitinate many proteins, recruiting specific autophagy‐related receptors to bind to LC3‐II to form autophagosomes. The proteins of BNIP3, NIX and FUNDC1, which are constituted and located in the outer membrane of mitochondria, can straightly adjust the phosphorylation of the LIR domain and combine with LC3‐II lipidation to form autophagosomes. Activation of these machineries can maintain and regulate the metabolism of this organelle, reactive oxidative species (ROS) production, biogenesis and mitochondrial DNA (mtDNA) damage repair.

### 2.2 Pathophysiology of stroke and MQC

#### 2.2.1 Pathophysiology of stroke

**FIGURE 1:**
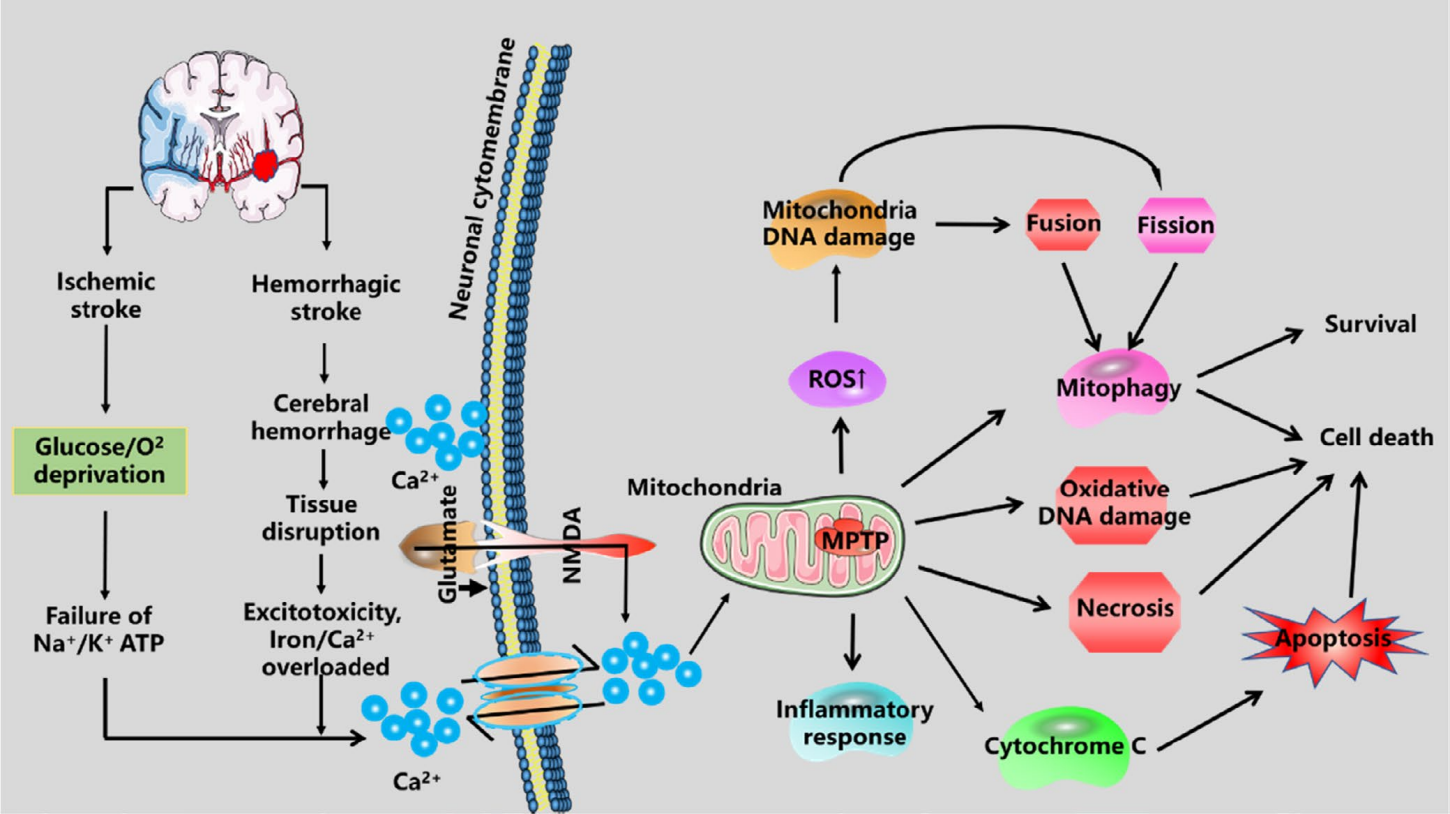
Overview of pathophysiology of stroke. Mitochondria plays an essential role in pathological conditions after ischaemic stroke and haemorrhagic stroke. During ischaemia, oxygen–glucose deprivation will cause ATP consumption and Na^+^/K^+^ATPase pump failure that induces depolarization of neuronal membranes and extreme release of glutamate. Excessive Ca^2+^ injection can induce ROS production and mitochondrial dysfunction including mitochondrial‐dependent division and fusion, mitochondrial‐induced apoptosis, as well as mitochondrial phagocytosis. These cellular processes ultimately lead to the death of neuron. During haemorrhage, haemorrhage in the brain parenchyma leads to the death of neuronal cells and releases lots of harmful substances, which damages the brain tissue. The injured nerve cells have ischaemia, hypoxia, acidosis and abnormal ion concentration, such as calcium iron overload

Stroke is the leading cause of adult mortality and disability in most developed and developing countries.^18^ As shown in Figure 1, many facts can influence the pathophysiological processes of stroke including energy disorders, cell acidosis, increased release of excitatory amino acids, intracellular calcium instability, free radical generation and apoptotic gene activation. These facts are causal and overlapping with each other, forming a vicious circle, and eventually cause apoptosis or necrosis.^19^

An ischaemic event occurs when blood flow to brain tissue is reduced or blocked. In patients with ischaemic stroke, a substantial drop in the focal cerebral blood flow can cause a lack of glucose and oxygen, which hastily impair biochemical effects, lead to cell death and ultimately brain damage.^20^ As is public with acute/traumatic injuries, the downstream signalling pathways of stroke can cause glutamate excitotoxicity and excessive calcium influx can induce ROS production and mitochondrial dysfunction.^21^ Mitochondrial dysfunction caused by oxygen and glucose deprivation (OGD) occurs in a few minutes after stroke, leading to consumption of ATP production and overproduction of ROS. The central area of ischaemia induces irreversible necrosis of neurons owing to interruption of blood supply and energy exhaustion. With respect to the ischaemic penumbra, glucose/energy metabolism disorder leads to a decrease in Na^+^/K^+^‐ATPase activity and triggers an imbalance of ion homeostasis, and then, cell membrane depolarization leads to a large amount of Ca^2+^ influx, and calcium overload leads to neurotransmitter valleys.

Haemorrhage stroke is a subtype of stroke which is related to high rates of disability and mortality.^22^ Mitochondria are very important in neuronal survival after haemorrhagic stroke. It accounts for 2,000,000 cases of haemorrhagic stroke worldwide each year, and those who survive usually have severe neurological debits.^23^ Presently, the typical opinion is that nerve injury after haemorrhagic stroke can be separated into secondary brain injury (SBI) and primary brain injury.^24^ The former is resulted from series of mechanisms containing inflammation, oxidative stress, neuronal death and mitochondrial dysfunction, whereas the latter is mainly induced by mechanical disruption after initial bleeding.^25^ Though most studies think that these mechanisms are associated with SBI after haemorrhagic stroke, effective interventions are still deficient.^26^ Therefore, it is imperative to discover ways of endorsing the recovery of nerve function in the treatment of haemorrhage stroke.

#### 2.2.2 Mitochondrial biogenesis in stroke

Mitochondrial biogenesis is defined as a process via which new mitochondria are formed by growth and division of pre‐existing mitochondria. This is the process that triggers an increase in mitochondrial mass. Mitochondrial biogenesis is related to the synthesis of the outer and inner mitochondrial membranes and mitochondrial encoded proteins; combination and imports of nuclear‐encoded mitochondrial proteins; and replication of mtDNA.^27^

**FIGURE 2:**
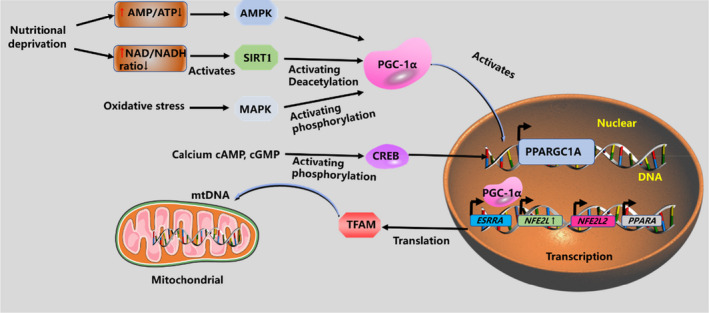
Overview of mitochondrial biogenesis. Different cell‐signalling pathways are activated, such as AMPK, SIRT1, MAPK and CREB, which are related to mitochondria biogenesis via increasing PGC‐1α gene transcription. PGC‐1α activates other transcription factors such as NRF1/2, which take charge of driving the transcription of nuclear‐encoded mitochondrial proteins, then, leading to increase expression of TFAM, driving transcription and replication of mtDNA

Mitochondrial biogenesis is tightly regulated by several cell‐signalling pathways. SIRT1‐PGC‐1a and AMPK‐PGC‐1a axes are two key pathways that adjust mitochondrial biogenesis (Figure 2).^27^ Calcium‐calcium/calmodulin‐dependent protein kinase (CaMK)‐Cyclic AMP response element‐binding protein (CREB), Akt‐CREB, PKA (protein kinase A)‐CREB and peroxisome proliferator‐activated receptor alpha (PPARa) ‐PGC‐1α pathways may also activate mitochondrial biogenesis. The target genes of PGC‐1α are nuclear respiratory factor 1 and nuclear respiratory factor 2 (scription and maintains the mitochondrial copy number).^28^ SIRT1 (silent information regulator 2 homolog 1), NRF1/2 and TFAM, are vital cell survival proteins particularly in oxidative stress environments, and SIRT1 is considered to be located in the nuclei, but in some cell types, it can also be knew to shuttle between cytoplasm and nuclear.^29^ Additionally, the mTOR signalling is tightly linked to mitochondria biogenesis, and activation of this pathway directly causes an increase in the expression of PGC‐1α. The underlying mechanism for the initial inhibition of proteolysis may be that phosphorylated E3‐ligase inhibits ubiquitination via mTORC1. The NAD/NADH ratio regulates Sirt1 activity, Calcium/CaMK activator, Caffeine activates mitochondrial biogenesis by increasing the concentration of catecholamines and increasing calcium flow, which activates AMPK upstream kinase calmodulin kinase.^30^ It is well known that endogenous nitric oxide triggers the transcription mechanism and drives the biogenesis of mitochondria. Besides, nitric oxide also induces vasodilation, which advances the availability of carbon substrate and oxygen for cell metabolism and respiration.^31^

Brain damage induced by haemorrhagic stroke leads to a decrease in cellular ATP, which leads to the phosphorylation of AMPK.^32^ As the critical downstream signal molecule of AMPK, transcriptional coactivator PGC‐1α arbitrates many mitochondrial functions (e.g. Δψm, ROS production and mitochondrial biogenesis).^33^ PGC‐1α could improve ATP production and mitochondrial mass in Alzheimer's disease and haemorrhagic stroke via activating NRF1/TFAM axis.^34^

#### 2.2.3 Mitochondrial autophagy in stroke

**FIGURE 3:**
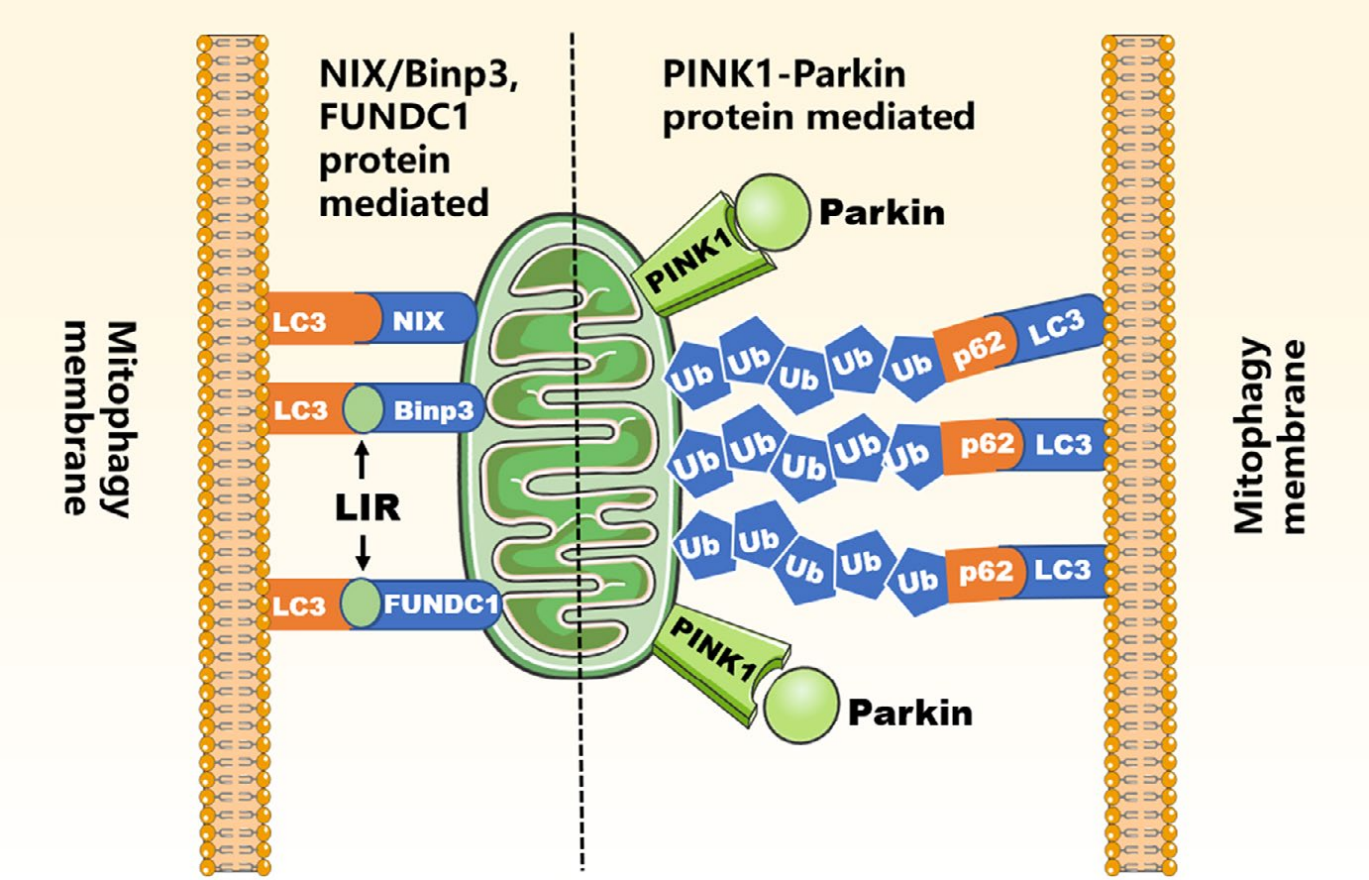
Overview of mitophagy. In dysfunctional mitochondria, FUNDC1 is located on the outer mitochondrial membrane and serves as a receptor for mitophagy under hypoxic conditions. PINK1 accumulates in the outer mitochondrial membrane. Nevertheless, BNIP3/Nix is located on the outer mitochondrial membrane and then serves as a mitophagy receptors, directly binding to the phagosome through LC3

Mitophagy, the selective autophagy of mitochondria, promotes the recovery of damaged mitochondrial components and controls oxidative stress.^35^ Mitochondria fine‐tune the biogenesis of mitochondria, and homeostasis plays a significant role in the physiology of cells and organisms. The imbalance between them may cause the accumulation of mitochondria, excessive ROS generation and attenuated oxygen consumption, which eventually causes cell degeneration and activation of cell death pathways.^36^ Mitophagy is mediated by many signalling pathways including the Parkin/PINK1 pathway, Bnip3, NIX and FUNDC1(Figure 3).

In the healthy mitochondria, PTEN‐induced PINK1 is constantly imported to the inner membrane, in which it directly determines the state of mitophagy.^37^ With respect to the mitochondrial damage, the stable and accumulated PINK1 on the OMM allows the kinase domain of PINK1 to phosphorylate OMM proteins, in addition to recruitment and activation of the E3‐ubiquitin ligase Parkin. In this case, once activating Parkin, it could polyubiquitinate proteins to the OMM. However, mutations in parkin would lead to ubiquitin proteasome system dysfunction.^38^ After cerebral ischaemia, Parkin protein depletion can increase the accumulation of ubiquitinated protein and the sensitivity of neurons to endoplasmic reticulum dysfunction.^39^

NIX and BNIP3 impact mitochondrial function in many methods and affect a series of mitochondrial and extramitochondrial functions.^40^ BNIP3 is protein that targets mitochondria and might induce mitochondrial damage. The research of Bnip3 in the brain largely focuses on apoptosis and necrotic cell death triggered by post‐ischaemic events.^41^ Yang et al. have indicated that BNIP3L/NIX may be a potential therapeutic target for ischaemic stroke, and BNIP3L/NIX may be in the mitochondria induced by cerebral ischaemia‐reperfusion (I‐R). Bnip3L can eliminate autophagy in mice and aggravate brain I‐R damage, which can be rescued by overexpression of BNIP3L.^42^ Hence, Bnip3/NIX is a promising target for controlling cell survival or death through regulating mitophagy after stroke.

FUNDC1 is a newly discovered mitophagy receptor that regulates the programmed eradication of mitochondria through unswervingly binding to LC3 under hypoxic states.^43^ The study has shown that tPA can repair mitochondrial function and decrease neuronal apoptosis by FUNDC1‐mediated mitophagy.^44^ Furthermore, the research has implied that rotenone can not only induce receptor‐mediated mitochondrial clearance, but also induce PINK1/Parkin‐dependent mitophagy for mitochondrial clearance, and that mitophagy has cytoprotective effects via removing damaged mitochondria, upon mitochondrial stress, the FUNDC1, mitophagy receptor regulates mitochondrial clearance. Lipidated LC3 (LC3II) expression increased, and FUNDC1 knockout cells showed a significant decrease in LC3 expression. Additionally, treatment of cells with autophagy flux inhibitor, chloroquine, induced further accumulation of LC3II, which indicated that rotenone‐induced mitophagy induced is owing to participation of mitochondrial FUNDC1.^45^

Ischaemic stroke is related to the activation or up‐regulation of mitophagy, more specifically, mitophagy pathways. Nevertheless, whether mitochondrial activation is a protective mechanism or in turn aggravates cell death is still the subject of ongoing research. The molecular mechanisms behind mitochondrial autophagy have made substantial progress. There is an agreement that up‐regulation of mitophagy during ischaemic stroke can provide protection.^46^

PINK1‐Parkin‐dependent mitochondrial maintenance is considered to depend on mitophagy. A landmark study showed Dendrobium nobile Lindl. Alkaloids impede manganese‐induced cytotoxicity, which may be mediated via modulating PINK1/Parkin‐mediated autophagic flux and advancing mitochondrial function.^47^ The study of investigating the underlying mechanism has shown that PINK1/Parkin‐mediated mitophagy was in a low‐temperature state in the slow rewarming group but was inhibited in the rapid‐rewarming group. Further inhibition of mitophagy in the slowly rewarming rats led to severe apoptosis, which highlighted the neuroprotective effect of PINK1/Parkin‐mediated mitophagy during slow rewarming after hypothermia.^48^ ROS is regarded as the cause of Parkin/PINK1‐dependent mitophagy. A research approved that ROS acts upstream of the PINK1/Parkin pathway to regulate mitophagy.^49^ Bcl‐2/adenovirus E1B 19‐kDainteracting protein 3 (Bnip3) is generally expressed in several cells and participates in a diversity of cell functions through participation in abundant cellular signalling pathways, including cell apoptosis, mitophagy and mitochondrial dysfunction.^50^ The study of Bnip3 in the brain largely focuses on apoptosis and necrotic cell death triggered by post‐ischaemic events.^51^ Shi et al. have shown that the deficiency of BNIP3 will suggestively attenuate both apoptosis and neuronal mitophagy but then will increase nonselective autophagy following ischaemic/hypoxic injury. The mitochondria‐localized BNIP3 networks with the autophagosome‐localized LC3, suggesting that BNIP3, similar to NIX, functions as an LC3‐binding receptor on mitochondria.^52^ The enhanced interaction between BNIP3 and LC3 may help induce excessive mitochondrial phagocytosis leading to cell death. Hence, Bnip3 is a promising target to operate cell survival or death via regulating mitophagy after ischaemic stroke. So far, the role of mitophagy in haemorrhagic stroke has not been wholly understood; however, further study is obliged to appreciate whether mitophagy is positive after haemorrhagic stroke.

#### 2.2.4 Mitochondrial fission and fusion in stroke

Mitochondria are highly dynamic cellular organelles characterized by their ability to change shape, size and position via highly harmonized measures of fission, fusion and transport to tactical locations. The basis of MQC is mitochondrial division/fusion.

**FIGURE 4:**
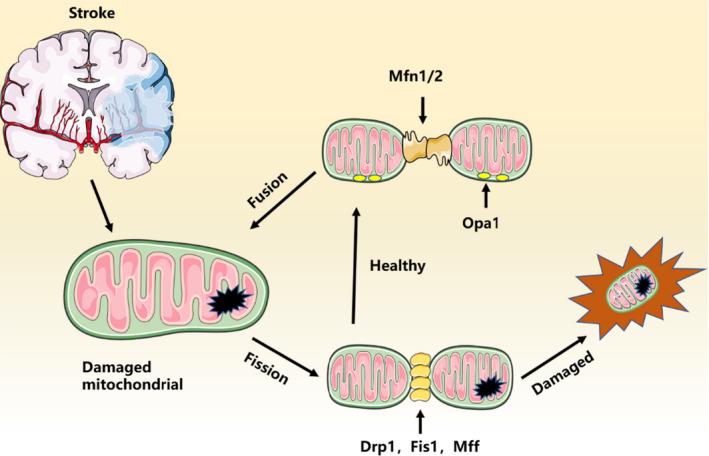
Relationship between mitochondrial fusion/fission in stroke

In the cell, mitochondria exist in a constantly changing dynamic situation, in which the mitochondrial network continuously extends and divides. Mitochondrial fission is primarily activated due to increased energy demand to hastily increase the number of mitochondria.^53^ Mitochondrial fission can eradicate dysfunctional mitochondria in the brain, and the degree of mitochondrial fission depends principally on the metabolic needs of cells. Appropriate mitochondrial fission produces many offspring, thereby promotes brain oxidative phosphorylation necessary for brain development and performance. Fission also permits mitochondria to separate impaired parts from reticular mitochondria, which is indispensable for the homeostasis of brain mitochondria. Fission of the mitochondria is a primary incident in apoptotic cell death following stroke.^54^ During stroke, mitochondrial division promotes the separation of damaged mitochondria to maintain the health of the entire mitochondria.^55^ The dynamic balance between the two determines the shape and structure of mitochondria. Mitochondrial division is mainly regulated by Drp1. Mitochondrial fission catalysed by Drp1 is necessary for mitochondrial biogenesis and maintenance of healthy mitochondria; Drp1 inhibition is neuroprotective. Drp1 is activated by dephosphorylation of an inhibitory phosphorylation site, Ser637 in ischaemic stroke.^56^ Drp1 is conveyed to the outer mitochondrial membrane through mitochondrial surface receptor Fis1\Mff.^57^ The broken link points on the mitochondria splits (Figure 4). Under homeostatic states, DRP1 is chiefly allocated in the cytoplasm. Nevertheless, in response to pathophysiological or physiological stimuli and changes in the concentration of ATP and calcium in cells, it promotes the transfer of DRP1 through protein kinase A (PKA) and calcineurin‐mediated post‐translational modifications to OMM.^58^ The study has indicated that the extramitochondrial kinase‐anchored protein 1 (AKAP1)/PKA complex constraints Drp1‐dependent mitochondrial fission, which can prevent neurons via keeping the activity of the respiratory chain, inhibiting superoxide production, as well as delaying Ca^2+^ deregulation. Drp1 inhibition may be of therapeutic relevance for the treatment of stroke and neurodegeneration.^59^

In contrast to mitochondrial fission, fusion is the process of integrating several mitochondrial parts into filamentous mitochondria. Mitochondrial fusion and mitochondrial network formation are connected with effective respiration and ATP production.^60^ Mitochondrial fusion is mainly regulated by mitochondrial fusion proteins (Mfn1,2) located in the outer mitochondrial membrane and optic atrophy 1, proteins Mfn1 and Mfn2, which contain two transmembrane domains in the OMM with a GTPase domain and are oriented towards the cytoplasm, Mfn1 and Mfn2, are required for efficient mitochondrial fusion.^61^ OPA1 situated in the inner mitochondrial membrane. The coiled‐coil spheres of Mfn1, 2 associate via interaction to form oligomer complexes and promote outer membrane fusion. OPA1 chiefly mediates mitochondrial inner membrane fusion. OPA1 is initiated in IMM and has the ability of GTPase related to power. At that moment, the mitochondrial lipid bilayer is mixed in a Mfn1/Mfn2‐dependent manner and undergoes GTP hydrolysis to offer energy to OMM fusion. Correspondingly, IMM fusion entails a similar action via OPA1 to permit to merge.^62^ Lai's group has reported that the stabilization of L‐OPA1 protects ischaemic brains via maintaining mitochondrial function and attenuating neuronal apoptosis. As a mitochondrial regulatory gene, OPA1 plays a dispensable role in the regulation of mitochondrial fission/fusion and other related functions, OPA1 as a talented therapeutic target for stroke prevention and treatment.^63^

Ischaemic stroke comprises a very complex physiopathology. The restoration of mitochondrial function may play an indispensable role in inhibiting the pathogenesis of stroke.^64^ The core reaction of mitochondrial fission is the compression and division of the two mitochondrial membranes. This response is caused by the recruitment of Drp1 to mitochondria via its receptor proteins, which are located in the outer membrane.^65^ Drp1 is lied in the surface of the outer mitochondrial membrane, a very insightful study has implied that Drp1 plays a crucial role in ischaemic stroke. Additionally, the infarct size is reduced after Drp1 is down‐regulated.^66^ A study has shown that inhibition of Drp1‐dependent mitochondrial fission via the outer mitochondrial AKAP1/PKA multifaceted prevents neurons from ischaemic stroke through preserving respiratory chain activity, inhibiting superoxide production, as well as delaying Ca^2+^ deregulation.^59^ Xu et al.^67^ have reported that YiQiFuMai inhibited mitochondrial apoptosis and activation of Drp1 in cerebral ischaemia‐injured rats, producing a substantial improvement in neurological score and cerebral infarction, as well as ameliorates ischaemic stroke‐induced neuronal apoptosis. Ischaemic stroke can tempt changes in mitochondrial morphology and function, OPA1, as a regulatory gene in mitochondria, plays a vital role in regulating mitochondrial fission/fusion and other relational functions, a study has indicated that the stabilization of long isoform of OPA1 (L‐OPA1) keeps ischaemic brains via maintaining mitochondrial function and attenuating neuronal apoptosis.^68^ The OPA1 can reduce brain oedema in ischaemic stroke, besides, its expression increased after exercise.^69^ Mitofusin 1 and 2 are homologous proteins, and both mediate mitochondrial fusions. A very insightful study has shown that the brain injury in ischaemic stroke rats can be resulted by the up‐regulation of mitochondrial E3 ubiquitin ligase 1. mitochondrial E3 ubiquitin ligase 1 leads to the interference of mitochondrial dynamics and function through the SUMOylating of Drp1 and the ubiquitination of Mfn2.^70^ A study in male rats have shown that this decreased Mfn‐2 expression might cause silenced or constrained fusion leading to mitochondrial fragmentation and upsurged sensitivity of mitochondria to apoptotic.^71^ Further study on the mechanism of mitochondrial dynamics is expected to provide a promising therapeutic target for the treatment and prevention of stroke.

In preventing haemorrhagic stroke, MQC mediated via Mfn1/2 and Drp1 has been indicated to play a pivotal role (eg brain oedema, inflammatory response and neural apoptosis).^72^ Earlier reports have implied that the Drp1 inhibitor mdivi1 can attenuate neuronal apoptosis and oxidative stress after subarachnoid haemorrhage.^73^ Moreover, current reports have indicated that Drp1 inhibitors exert neuroprotective effects in the haemorrhagic stroke models,^72^ suggesting that excessive Drp1 activity may be a significant risk factor for haemorrhagic stroke‐induced brain injury. Actually, mitochondrial damage tempted through acrolein around the hematoma after haemorrhagic stroke is linked to excessive mitochondrial fission and increased Drp1 translocation, and acrolein‐scavenging agents can pointedly impede Drp1‐mediated nuclear fission after haemorrhagic stroke as well as diminish mitochondrial morphological damage.^72^ First and foremost, inhibition of mitochondrial fission suggestively improves cerebral oedema, neurological deficits and neuronal apoptosis after haemorrhagic stroke.^74^

## 3 PHARMACOLOGY TARGETING MQC IN STROKE

### 3.1 Drug targeting mitochondrial biogenesis in stroke

Since mitophagy is usually also activated or blocked by pathologic processes, pharmacological methods for these pathways have attracted substantial interest.^75^ More and more studies have shown that the potential mechanism of drug prevention and treatment of stroke injury may be related to intervention in MQC. As shown in Table 1. Studies have shown that the ligustrazine modified compound TN‐2, the main component of the traditional Chinese medicine Ligusticum chuanxiong, has a protective effect on the damage of SH‐SY5Y and CGNs nerve cells induced by tetrazine pyridine, and can increase the mRNA of mitochondrial biosynthesis factors PGC‐1α and TFAM expression and promote mitochondrial biosynthesis.^76^ Morphine increased the expression of PGC‐1α and reduced the expression of cocaine‐amphetamine regulated transcript (CART). Nevertheless, Neuro Aid increased the expression of PGC‐1α, TFAM, CART and ΔfosB. Neuro Aid reinstated the effect of morphine on the expression of PGC‐1α and CART. The effect of Neuro Aid on morphine‐induced memory impairment/gene expression may be connected with its neuroprotective effects and anti‐apoptotic.^77^ Coincidentally, YiqiHuoxue Fang Danqi Capsules have also been found to inhibit morphine‐induced nerve damage and apoptosis, upregulate the gene expression of TFAM and PGC‐1α in damaged hippocampus and promote neuronal biosynthesis.^78^ Another study found that the flavonoid rutin in Tianshan Saussurea, a traditional Chinese medicine for warming yang, promoting blood circulation and replenishing qi, has a better anti‐fatigue effect on mice models of ‘qi deficiency’ induced by forced swimming, and relieves anxiety and neuroprotection driven by cerebral hypoxia. This is related to rutin can upsurge the expression of PGC‐1α and SIRT1 mRNA11/63.^79^ Stimulation of ETB receptors in a rat model of cerebral ischaemia with sovateltide is both neuroprotective and neuroregenerative. Sovateltide alleviates mitochondrial dysfunction via promoting biogenesis.^80^ Furthermore, one study has shown that Mangiferin is the predominant compound of extracts of Mangifera indica L and Anemarrhena asphodeloides L. Mangiferin treatment is accompanied via amelioration of mitochondrial dysfunction, oxidative stress and apoptosis, which might significantly upregulate the expressions of SIRT1 and PGC‐1α.^81^

**TABLE 1:** Drugs or compounds targeting mitochondrial biogenesis, mitophagy and dynamics‐fusion/fission in regulating stroke function

	Name	Targets	Functions	Effects	References
Mitochondrial biogenesis	The Novel Tetramethylpyrazine Bis‐nitrone (TN‐2)	PGC‐1α	Anti‐apoptotic promote mitochondrial biosynthesis	Increasing the mRNA of mitochondrial biosynthesis factors PGC‐1α and TFAM expression and promoting mitochondrial biosynthesis	76
	NeuroAid	PGC‐1α	Inducing neuroprotective and anti‐apoptotic	NeuroAid restored the effect of morphine on the expression of PGC‐1α and CART	77
	YiqiHuoxue Fang Danqi Capsules	PGC‐1α	Inducing neuroprotective and anti‐apoptosis	Up‐regulation gene expression of TFAM and PGC‐1α in damaged hippocampus and promoting neuronal biosynthesis	78
	Rutin	PGC‐1α SIRT1	Anti‐fatigue, relieves anxiety and neuroprotection	Promoting blood circulation and replenishing qi, anti‐fatigue and relieves anxiety and neuroprotection caused by cerebral hypoxia	79
	Sovateltide	PGC‐1α	Anti‐apoptosis	Sovateltide promotes mitochondrial biogenesis along with promoting neural progenitor cell mediated neuronal regeneration after stroke	80
	Mangiferin	PGC‐1α SIRT1	Anti‐oxidative, anti‐apoptosis, and anti‐inflammation	Up‐regulation the expressions of SIRT1 and PGC‐1α, amelioration of mitochondrial dysfunction, oxidative stress, and apoptosis	81
Mitochondrial mitophagy	Danhong injection	Parkin	Anti‐apoptosis	Up‐regulation Parkin expression, ameliorating mitochondrial dysfunction and neuron apoptosis	82
	Naringin	Parkin	Anti‐apoptosis, anti‐oxidative, anti‐inflammatory	Decreasing the ratio of LC3‐II to LC3‐I in mitochondrial fraction, and inhibiting the translocation of Parkin to the mitochondria in the ischaemia‐reperfused brains	83
	Rehmapicroside	Drp1/ PINK1/ Parkin	Anti‐apoptosis, inhibiting mitophagy reducing infarct sizes	Up‐regulated Bcl‐2 but down‐regulated Bax, Caspase‐3 and cleaved Caspase‐3, and down‐regulated PINK1, Parkin, p62 and the ratio of LC3‐II to LC3‐I in the OGD/RO‐treated PC12 cells	84
	Xiao‐Xu‐Ming Decoction	LC3	Suppressed mitophagy activation, and reduced mitophagy	Down‐regulating the expression levels of LC3, Beclin1, Lamp1 and mitochondrial p62, inhibiting excessive mitochondrial autophagy and protecting cerebral ischaemia‐reperfusion injury	85
	Garciesculenxanthone B (GeB)	PINK1‐Parkin	Anti‐inflammatory, antioxidative, and neuroprotective activities	GeB can stabilize PINK1 and induce mitophagy via the PINK1–Parkin‐dependent pathway. GeB has a neuroprotective effect	86
	Pinocembrin	LC3	Antioxidant and anti‐inflammatory and antimicrobial	Decreasing the expression of LC3II and Beclin1 and increased the level of p62 up‐regulated autophagy	87
	Sodium Tanshinone IIA Sulfonate	SIRT6	Anti‐inflammatory inhibited of autophagy	Up‐regulation of autophagy associated proteins, such as LC3‐II, Beclin‐1 and Sirt 6	88
	Compound K	AMPK	Neuroprotective anti‐apoptosis inhibited of autophagy	Increasing cell viability and decreasing the ROS generation	89
	Geniposide	NLRP3	Anti‐inflammatory inhibited of autophagy	Down‐regulating the NLRP3 inflammasome and promotion of autophagy activation	90
	Y RapaLink‐1	LC3 and Beclin‐1	Anti‐apoptosis and enhanced autophagy	RapaLink‐1 prevented cell apoptosis and enhanced autophagy of macrophages	91
	Neuroprotectin D1	RNF146and Wnt/β‐catenin	Neuroprotective and anti‐inflammatory	Upregulating RFP146 and activating Wnt/b‐catenin pathway	92
	Mu‐Xiang‐You‐Fang	AMPK/mTOR	Anti‐inflammatory, anti‐apoptotic, and anti‐oxidative stress	Inhibited autophagy after OGD/R‐induced PC12 cell injury	93
	Eugenol	AMPK/mTOR	Anti‐apoptosis neuroprotective	Promoted autophagy and OGD/R‐induced autophagy was strengthened by eugenol	94
Mitochondrial dynamics‐fusion/split	Atractylenolide III	Drp1	Anti‐oxidative and anti‐inflammatory	A III reduced Drp1 phosphorylation, translocation and prevented mitochondrial fission	95
	Baicalin	Drp1	Anti‐apoptosis reduces ROS production in vitro	Baicalin reduces ROS production in vitro, prevents mitochondrial fission, and promotes Drp‐1 phosphorylation (Ser637) and regulates Drp‐1 and MFN2 expression in PC12 cells	96
	Ginkgolide K	Drp1	Anti‐apoptosis reduces ROS production	GK reduces mitochondrial ROS production and suppresses mitochondrial fission in N2a cells and attenuates Drp1 and GSK‐3β translocation to mitochondria after I/R injury in vivo	97
	Neuroprotection of hydroxysafflor yellow A(HSYA)	Drp1	Inhibit cell apoptosis reduce ROS levels	Reduced the expression of the mitochondrial fission protein DRP1	98
	Esculetin	Drp1	Anti‐oxidative and anti‐inflammatory	Inhibiting mitochondrial apoptosis, up‐regulation Nrf‐2 and increasing the expression of Drp1	99
	LyciumBarbarum Polysaccharides	Drp‐1 and Opa1	Neuroprotective	Increasing the expression of the mitochondrial fusion factor, Opa1, and decrease the expression of the mitochondrial cleavage factor, Drp1	100
	DL‐3‐n‐Butylphthalide	JNK, p38	Anti‐apoptosis	Reduced ischaemia‐induced intracellular Ca^2+^ accumulation, inflammation, lipid peroxidation, and superoxide radical formation	101

### 3.2 Drug targeting mitochondrial dynamics in stroke

Mitochondrial division and fusion are sensitive to drugs. Therefore, the realization of targeted drug delivery is very pivotal for MQC. At present, many studies have focused on this aspect. FufangDanhong injection can enhance the expression of parkin protein in the cerebral ischaemic brain and increase the relative mitochondrial reductase activity of neurons cultured in vitro under OGD.^82^ Naringin attenuated infarct size, decreased apoptotic cell death and reduced neurological deficit score in the ischaemia‐reperfused rat brains. Besides, naringin reduced the ratio of LC3‐II to LC3‐I in mitochondrial fraction, attenuated 3‐nitrotyrosine formation and restrained the translocation of Parkin to the mitochondria.^83^ Rehmanniaehas neuroprotective effects on attenuating infarct sizes, decreasing apoptotic cell death, curbing mitophagy and enhancing neurological functions during cerebral ischaemia‐reperfusion injury.^84^ Xiao‐Xu‐Ming Decoction can play a neuroprotective effect via down‐regulating the expression levels of LC3, Beclin1, Lamp1 and mitochondrial p62, thereby inhibiting excessive mitochondrial autophagy and protecting cerebral ischaemia‐reperfusion injury.^85^ Garciesculenxanthone B (GeB), a kind of xanthones extracted from mountain papaya, can partially rescue the brain damage induced by ischaemia‐reperfusion in mice, and can stabilize the changes in the content of PINK1 in brain tissues driven by cerebral ischaemia, which riggers Parkin to translocate to damaged mitochondria to induce mitosis. These effects are eliminated by knocking out the PINK1 gene^86^. Pinocembrin could remarkably decrease the expression of LC3II and Beclin1 and increase the level of p62 in hippocampus CA1 of I/R rats. Furthermore, Pinocembrin also could decrease RAPA‐induced excessive activation of autophagy and neuronal damage in I/R rats.^87^ A previous report showed that Sodium tanshinone IIA sulfonate treatment reduced neuroinflammation and reduced the up‐regulation of autophagy associated proteins,^88^ and a report has shown that pretreatment with Compound K (CK) prevented neurons from OGD/R injury via decreasing the ROS generation and increasing cell capability, Ca^2+^ overload and mitochondrial damage. Additionally, CK reduces autophagy‐mediated apoptosis via boosting the process of forming autophagosomes into phagocytic precursors.^89^ A research implied that geniposide substantially diminished inflammatory response in BV‐2 microglial cells after OGD/R. The effect of geniposide may be due to the reduction of the levels of inflammatory cytokines through restraining the activation and expression of NLR family pyrin domain‐containing 3 (NLRP3) inflammasome as well as increasing autophagic activity following OGD/R in BV‐2 microglial cells.^90^ A study revealed that RapaLink‐1 prevented cell apoptosis and enhanced autophagy of macrophages revealed and inhibited the formation of thrombus plaque.^91^ Neuroprotectin D1 (NPD1) is a docosahexaenoic acid derivative with anti‐inflammatory and neuroprotective properties. NPD1 impeded I/R‐induced excessive autophagy via upregulating RFP146 and activating Wnt/b‐catenin pathway.^92^ Mu‐Xiang‐You‐Fang could significantly increase cell viability and mitochondrial membrane potential and decrease the calcium concentration and inhibit the autophagy induced by OGD/R and inhibited the expression of LC3, beclin1, p‐AMPK and ULK1, it can protect cerebral OGD/R injury.^93^ Induced autophagy through the mammalian target of AMPK/rapamycin (mTOR)/P70S6K signalling pathway. Eugenol pretreatment can reduce brain I/R damage.^94^

### 3.3 Drug targeting mitophagy in stroke

The restoration of mitochondrial autophagy is very vital for regulating the homeostasis of mitochondria. At the same time, it has a great effect on preventing stroke and achieving cell recovery after stroke. Many targeted therapies have been developed to trigger the restoration of autophagy. Atractylenolide III can promote mitochondrial function recovery and biogenesis via regulating the mitochondrial fission protein Drp1, antagonize OGD/R‐induced mitochondrial damage caused by ROS, thereby exerting neuroprotective effects.^95^ Baicalin alleviates cerebral I/R‐induced brain injury exacerbated by hyperglycaemia in rats, prevents mitochondrial fission by regulating the expression of Drp‐1 and MFN2, and the phosphorylation of Drp‐1 (Ser637). In addition, baicalin treatment can prevent apoptosis and promote mitophagy.^96^ Ginkgolides are natural antagonists of platelet‐activating factor receptor, which can protect neuronal function via attenuating oxidative stress and inflammation after I/R injury. Treatment of mice with Ginkgolide K can prevent GSK‐3β and Drp1 translocation to mitochondria and reduce mitochondrial dysfunction after middle cerebral artery occlusion.^97^ A study has suggested that I/R and OGD/R stress increased the levels of phenylalanine, impaired mitochondrial function and induced ROS production. However, HSYA could reduce phenylalanine levels and promote mitochondrial function via the up‐regulation of mitochondrial fission protein DRP1.^98^ The pharmacological mechanism of Esculetin involves its effect on mitochondrial autophagy. In addition, apoptosis triggered by mitochondrial oxidative stress mediated by mitochondrial fragments during transient cerebral ischaemia and reperfusion injury. Esculetin treatment effectively increased the expression of Drp1. Studies have suggested that mitochondrial regulatory pathways play a crucial role in the pathological process of cerebral ischaemia, besides, Esculetin‐mediated increase in mitochondrial autophagy and decrease in mitochondrial fragmentation as well as apoptosis play a protective role in transient cerebral ischaemia and reperfusion injury.^99^ In a rat model of transient focal cerebral ischaemia/reperfusion, diabetic hyperglycaemia down‐regulates OPA1 and up‐regulates Drp1. LyciumBarbarum Polysaccharides intervention can increase the expression of mitochondrial fusion factor OPA1 and decrease the expression of mitochondrial lysis factor Drp1.^100^ Studies have indicated that DL‐3‐n‐Butylphthalide prevents ischaemic injury through multiple mechanisms, including mitochondrial‐related caspase‐dependent and independent apoptotic pathways. This research encourages the exploration of DL‐3‐n‐Butylphthalide as a neuroprotective drug which is used to treat ischaemic stroke.^101^

Regardless of the diversity of obtainable therapeutic choices, owing to the short efficacy of neuroprotective drugs high prevalence rates of stroke are still recorded. Therefore, novel alternative therapeutic strategies are urgently needed to decrease the incidence. Furthermore, to treat stroke clinically, mitochondrial targeting through pharmacological mediators is still a challenge.

## 4 CONCLUSIONS AND PERSPECTIVES

Mitochondrial dysfunction plays vital role in attenuating the devastating consequences of stroke, especially linking to targeting mitochondria and the pathology of stroke via new therapeutic strategies. This advances the prospective of treating stroke with innovative mitochondria‐targeted therapies. The cell death is usually caused via decamped bioenergetics, abnormal mitochondrial morphology, and structure, as well as uncharacteristic mitochondrial fission/fusion. In this sense, MQC can be an efficacious way of curing stroke.^102^

In this review, we offer an inclusive portrayal of the involvement of MQC in the ischaemic stroke and haemorrhagic stroke. Notably, MQC exerts a vital role in the pathophysiological procedure of stroke development and provides reason for further exploration in stroke. MQC is not only involved in regulating cell metabolism divert but also involved in energy metabolism. The enhancement of MQC can be realized by the pharmacological initiation of mitochondrial biogenesis, mitophagy and mitochondrial fission/fusion. These methods are emerging as new strategy for the prevention or treatment of stroke. As indispensable regulators of MQC, mitochondrial dynamics and mitophagy play a vital role in keeping mitochondrial homeostasis in healthy cells. Defects in MQC have also been involved in contributing to stroke.

At present, research on MQC in stroke is still in its insufficient phase. Preclinical studies suggest that targeting MQC by genetic interventions or pharmacological features is neuroprotective.^103^ Mitochondrial targeting via pharmacological agents is still inspiring in the clinic. It is reported that the adjacent cells will save the impaired cells when the injured mitochondria receive the different extracellular stimuli from outer space. Hence, further research is required to clarify the mechanisms behind the functions of MQC in ischaemic stroke and haemorrhagic stroke and provide new insights, for instance accurate timing and new target molecules. Here, we conclude that the future study on MQC can be conducted in the following directions. AMPK is the core factor of the MQC mechanism, however, whether AMPK exists in mitochondria is still unknown. In view of the fact that mitochondria are an indispensable place for energy production in the body, and AMPK is a sensor that is extremely sensitive to energy changes, it is speculated that AMPK may be located in mitochondria, but this hypothesis urgently needs further research to confirm.^104^ PINK1/Parkin has been proven to be the most effective way to regulate mitochondrial autophagy, but it is not clear whether AMPK and its downstream target genes participate in the regulation of PINK1/Parkin pathway. It is urgent to advance new drugs for curing stroke. Although there are still many blanks in the current research on stroke and MQC, it is undeniable that the solution of the above problems will help humans to fully understand the molecular mechanism of MQC in stroke and provide a theoretical basis for future treatment.

## CONFLICTS OF INTEREST

The authors declare that they have no conflicts of interest.

## AUTHOR CONTRIBUTION

**Heyan Tian:** Writing – original draft (equal); Writing – review & editing (equal). **Xiangyu Chen:** Conceptualization (supporting); Software (supporting); Writing – original draft (supporting); Writing – review & editing (supporting). **Jun Liao:** Conceptualization (supporting); Funding acquisition (equal); Validation (supporting); Writing – review & editing (supporting). **Tong Yang:** Conceptualization (supporting); Writing – review & editing (supporting). **Shaowu Cheng:** Conceptualization (supporting); Writing – review & editing (supporting). **Zhigang Mei:** Conceptualization (supporting); Funding acquisition (lead); Investigation (lead); Project administration (lead); Writing – review & editing (supporting). **Jinwen Ge:** Conceptualization (supporting); Funding acquisition (equal); Project administration (supporting); Writing – review & editing (supporting).

## References

[1] Wu S , Wu B , Liu M , et al. Stroke in China: advances and challenges in epidemiology, prevention, and management. Lancet Neurol. 2019;18(4):394‐405. 30878104 10.1016/S1474-4422(18)30500-3

[2] Zhou M , Wang H , Zeng X , et al. Mortality, morbidity, and risk factors in China and its provinces, 1990–2017: a systematic analysis for the Global Burden of Disease Study 2017. Lancet. 2019;394(10204):1145‐1158. 31248666 10.1016/S0140-6736(19)30427-1 PMC6891889

[3] Wang W , Jiang B , Sun H , et al. Prevalence, incidence, and mortality of stroke in China: results from a nationwide population‐based survey of 480 687 adults. Circulation. 2017;135(8):759‐771. 28052979 10.1161/CIRCULATIONAHA.116.025250

[4] Amarenco P , Bogousslavsky J , Caplan LR , Donnan GA , Hennerici MG . Classification of stroke subtypes. Cerebrovasc Dis. 2009;27(5):493‐501. 19342825 10.1159/000210432

[5] Ikram MA , Wieberdink RG , Koudstaal PJ . International epidemiology of intracerebral hemorrhage. Curr Atheroscler Rep. 2012;14(4):300‐306. 22538431 10.1007/s11883-012-0252-1 PMC3388250

[6] D'Arcy MS . Cell death: a review of the major forms of apoptosis, necrosis and autophagy. Cell Biol Int. 2019;43(6):582‐592. 30958602 10.1002/cbin.11137

[7] Henderson SJ , Weitz JI , Kim PY . Fibrinolysis: strategies to enhance the treatment of acute ischemic stroke. J Thromb Haemost. 2018;16(10):1932‐1940. 29953716 10.1111/jth.14215

[8] Morgenstern LB , Hemphill JC 3rd , Anderson C , et al. Guidelines for the management of spontaneous intracerebral hemorrhage: a guideline for healthcare professionals from the American Heart Association/American Stroke Association. Stroke. 2010;41(9):2108‐2129. 20651276 10.1161/STR.0b013e3181ec611b PMC4462131

[9] Li X , Huang X , Tang Y , et al. Assessing the pharmacological and therapeutic efficacy of traditional Chinese medicine liangxue tongyu prescription for intracerebral hemorrhagic stroke in neurological disease models. Front Pharmacol. 2018;9:1169. 30459599 10.3389/fphar.2018.01169 PMC6232344

[10] Gao F , Zhang J . Mitochondrial quality control and neurodegenerative diseases. Neuronal Signal. 2018;2(4):NS20180062. 32714594 10.1042/NS20180062 PMC7373240

[11] Qiu Z , Wei Y , Song Q , et al. The role of myocardial mitochondrial quality control in heart failure. Front Pharmacol. 2019;10:1404. 31866862 10.3389/fphar.2019.01404 PMC6910121

[12] Roque W , Cuevas‐Mora K , Romero F . Mitochondrial quality control in age‐related pulmonary fibrosis. Int J Mol Sci. 2020;21(2):643. 10.3390/ijms21020643 PMC7013724 31963720

[13] Tang BL . Sirt1 and the mitochondria. Mol Cells. 2016;39(2):87‐95. 26831453 10.14348/molcells.2016.2318 PMC4757807

[14] Watters O , Connolly NMC , Konig H‐G , Dussmann H , Prehn JHM . AMPK preferentially depresses retrograde transport of axonal mitochondria during localized nutrient deprivation. J Neurosci. 2020;40(25):4798‐4812. 32393534 10.1523/JNEUROSCI.2067-19.2020 PMC7326360

[15] Summer R , Shaghaghi H , Schriner D , et al. Activation of the mTORC1/PGC‐1 axis promotes mitochondrial biogenesis and induces cellular senescence in the lung epithelium. Am J Physiol Lung Cell Mol Physiol. 2021;320(3):L468‐L471. 30892080 10.1152/ajplung.00244.2018 PMC6620667

[16] Benard G , Karbowski M . Mitochondrial fusion and division: regulation and role in cell viability. Semin Cell Dev Biol. 2009;20(3):365‐374. 19530306 10.1016/j.semcdb.2008.12.012 PMC2768568

[17] Ma K , Chen G , Li W , Kepp O , Zhu Y , Chen Q . Mitophagy, mitochondrial homeostasis, and cell fate. Front Cell Dev Biol. 2020;8:467. 32671064 10.3389/fcell.2020.00467 PMC7326955

[18] Guzik A , Bushnell C . Stroke epidemiology and risk factor management. Continuum (Minneap Minn). 2017;23(1):15‐39. 28157742 10.1212/CON.0000000000000416

[19] Li Y , Zhang X , Ma A , Kang Y . Rational application of beta‐hydroxybutyrate attenuates ischemic stroke by suppressing oxidative stress and mitochondrial‐dependent apoptosis via activation of the Erk/CREB/eNOS pathway. ACS Chem Neurosci. 2021;12(7):1219‐1227. 33739811 10.1021/acschemneuro.1c00046

[20] Yang J‐L , Mukda S , Chen S‐D . Diverse roles of mitochondria in ischemic stroke. Redox Biol. 2018;16:263‐275. 29549824 10.1016/j.redox.2018.03.002 PMC5854930

[21] Galluzzi L , Kepp O , Kroemer G . Mitochondria: master regulators of danger signalling. Nat Rev Mol Cell Biol. 2012;13(12):780‐788. 23175281 10.1038/nrm3479

[22] Feigin: update on the global burden of ischemic and hemorrhagic stroke in 1990‐2013: the GBD 2013 Study. Neuroepidemiology. 2015;45(3):161–176. 26505981 10.1159/000441085 PMC4633282

[23] Kumar S , Selim M , Marchina S , Caplan LR . Transient neurological symptoms in patients with intracerebral hemorrhage. JAMA Neurol. 2016;73(3):316‐320. 26747699 10.1001/jamaneurol.2015.4202

[24] Aronowski J , Zhao X . Molecular pathophysiology of cerebral hemorrhage secondary brain injury. Stroke. 2011;42(6):1781‐1786. 21527759 10.1161/STROKEAHA.110.596718 PMC3123894

[25] Yu J , Zheng J , Lu J , Sun Z , Wang Z , Zhang J . AdipoRon protects against secondary brain injury after intracerebral hemorrhage via alleviating mitochondrial dysfunction: possible involvement of AdipoR1‐AMPK‐PGC1 pathway. Neurochem Res. 2019;44(7):1678‐1689. 30982205 10.1007/s11064-019-02794-5

[26] Cordonnier C , Demchuck A , Ziai W , Anderson CS . Intracerebral haemorrhage: current approaches to acute management. Lancet. 2018;392(10154):1257–1268. 30319113 10.1016/S0140-6736(18)31878-6

[27] Li PA , Hou XL , Hao SC . Mitochondrial biogenesis in neurodegeneration. J Neurosci Res. 2017;95(10):2025‐2029. 28301064 10.1002/jnr.24042

[28] Farge G , Falkenberg M . Organization of DNA in mammalian mitochondria. Int J Mol Sci. 2019;20(11):2770. 10.3390/ijms20112770 PMC6600607 31195723

[29] Yanagisawa S , Baker JR , Vuppusetty C , et al. The dynamic shuttling of SIRT1 between cytoplasm and nuclei in bronchial epithelial cells by single and repeated cigarette smoke exposure. PLoS One. 2018;13(3):e0193921. 29509781 10.1371/journal.pone.0193921 PMC5839577

[30] Ruderman NB , Xu XJ , Nelson L , et al. AMPK and SIRT1: a long‐standing partnership? Am J Physiol Endocrinol Metab. 2010;298(4):E751‐E760. 20103737 10.1152/ajpendo.00745.2009 PMC2853213

[31] Suliman HB , Piantadosi CA . Mitochondrial biogenesis: regulation by endogenous gases during inflammation and organ stress. Curr Pharm Des. 2014;20(35):5653‐5662. 24606800 10.2174/1381612820666140306095717 PMC4276344

[32] Mukherjee P , Mulrooney TJ , Marsh J , Blair D , Chiles TC , Seyfried TN . Differential effects of energy stress on AMPK phosphorylation and apoptosis in experimental brain tumor and normal brain. Mol Cancer. 2008;7(1):37. 18474106 10.1186/1476-4598-7-37 PMC2397440

[33] Handschin C , Spiegelman BM . Peroxisome proliferator‐activated receptor coactivator 1 coactivators, energy homeostasis, and metabolism. Endocr Rev. 2007;27(7):728‐735. 10.1210/er.2006-0037 17018837

[34] You Y , Hou YH , Zhai X , et al. Protective effects of PGC‐1 alpha via the mitochondrial pathway in rat brains after intracerebral hemorrhage. Brain Res. 2016;1646:34‐43. 27178364 10.1016/j.brainres.2016.04.076

[35] Bhatia D , Choi ME . The emerging role of mitophagy in kidney diseases. J Life Sci (Westlake Village). 2019;1(3):13‐22. 32099974 10.36069/jols/20191203 PMC7041910

[36] Khalifa ARM , Abdel‐Rahman EA , Mahmoud AM , et al. Sex‐specific differences in mitochondria biogenesis, morphology, respiratory function, and ROS homeostasis in young mouse heart and brain. Physiol Rep. 2017;5(6):e13125. 28325789 10.14814/phy2.13125 PMC5371549

[37] Greene AW , Grenier K , Aguileta MA , et al. Mitochondrial processing peptidase regulates PINK1 processing, import and Parkin recruitment. EMBO Rep. 2012;13(4):378‐385. 22354088 10.1038/embor.2012.14 PMC3321149

[38] Cook C , Stetler C , Petrucelli L . Disruption of protein quality control in Parkinson's disease. Cold Spring Harb Perspect Med. 2012;2(5):a009423. 22553500 10.1101/cshperspect.a009423 PMC3331692

[39] Mengesdorf T , Jensen PH , Mies G , Aufenberg C , Paschen W . Down‐regulation of parkin protein in transient focal cerebral ischemia: a link between stroke and degenerative disease? Proc Natl Acad Sci USA. 2002;99(23):15042‐15047. 12415119 10.1073/pnas.232588799 PMC137541

[40] Ney PA . Mitochondrial autophagy: origins, significance, and role of BNIP3 and NIX. Biochim Biophys Acta Mol Cell Res. 2015;1853(10):2775‐2783. 10.1016/j.bbamcr.2015.02.022 25753537

[41] Shao Z , Dou S , Zhu J , et al. The role of mitophagy in ischemic stroke. Front Neurol. 1809;2020:11. 10.3389/fneur.2020.608610 PMC7793663 33424757

[42] Yuan Y , Zheng Y , Zhang X , et al. BNIP3L/NIX‐mediated mitophagy protects against ischemic brain injury independent of PARK2. Autophagy. 2017;13(10):1754‐1766. 28820284 10.1080/15548627.2017.1357792 PMC5640199

[43] Li W , Zhang X , Zhuang H , et al. MicroRNA‐137 is a novel hypoxia‐responsive microRNA that inhibits mitophagy via regulation of two mitophagy receptors FUNDC1 and NIX. J Biol Chem. 2014;289(15):10691‐10701. 24573672 10.1074/jbc.M113.537050 PMC4036186

[44] Cai Y , Yang E , Yao X , et al. FUNDC1‐dependent mitophagy induced by tPA protects neurons against cerebral ischemia‐reperfusion injury. Redox Biol. 2021;38:101792. 33212415 10.1016/j.redox.2020.101792 PMC7679257

[45] Park SY , Koh HC . FUNDC1 regulates receptor‐mediated mitophagy independently of the PINK1/Parkin‐dependent pathway in rotenone‐treated SH‐SY5Y cells. Food Chem Toxicol. 2020;137:111163. 32001317 10.1016/j.fct.2020.111163

[46] Sciarretta S , Maejima Y , Zablocki D , Sadoshima J . The role of autophagy in the heart. Annu Rev Physiol. 2018;80(80):1‐26. 29068766 10.1146/annurev-physiol-021317-121427

[47] Fu X , Chen S , Wang X , et al. Dendrobium nobile Lindl. alkaloids alleviate Mn‐induced neurotoxicity via PINK1/Parkin‐mediated mitophagy in PC12cells. Biochem Biophys Rep. 2021;26:100877. 33889759 10.1016/j.bbrep.2020.100877 PMC8047462

[48] Hu Y , Sun D , Li Y , et al. Increased PINK1/Parkin‐mediated mitophagy explains the improved brain protective effects of slow rewarming following hypothermia after cardiac arrest in rats. Exp Neurol. 2020;330:113326. 32330551 10.1016/j.expneurol.2020.113326

[49] Wei X , Qi Y , Zhang X , et al. ROS act as an upstream signal to mediate cadmium‐induced mitophagy in mouse brain. Neurotoxicology. 2015;46:19‐24. 25464205 10.1016/j.neuro.2014.11.007

[50] Yuan C , Pu LQ , He ZL , Wang J . BNIP3/Bcl‐2‐mediated apoptosis induced by cyclic tensile stretch in human cartilage endplate‐derived stem cells. Exp Ther Med. 2018;15(1):235‐241. 29375685 10.3892/etm.2017.5372 PMC5763692

[51] Xiao B , Goh J‐Y , Xiao L , Xian H , Lim K‐L , Liou Y‐C . Reactive oxygen species trigger Parkin/PINK1 pathway‐dependent mitophagy by inducing mitochondrial recruitment of Parkin. J Biol Chem. 2017;292(40):16697‐16708. 28848050 10.1074/jbc.M117.787739 PMC5633131

[52] Zhu Y , Massen S , Terenzio M , et al. Modulation of serines 17 and 24 in the LC3‐interacting region of Bnip3 determines pro‐survival mitophagy versus apoptosis. J Biol Chem. 2013;288(2):1099‐1113. 23209295 10.1074/jbc.M112.399345 PMC3542995

[53] Kanaan GN , Ichim B , Gharibeh L , et al. Glutaredoxin‐2 controls cardiac mitochondrial dynamics and energetics in mice, and protects against human cardiac pathologies. Redox Biol. 2018;14:509‐521. 29101900 10.1016/j.redox.2017.10.019 PMC5675898

[54] Jia J , Jin H , Nan D , Yu W , Huang Y . New insights into targeting mitochondria in ischemic injury. Apoptosis. 2021;26(3–4):163‐183. 33751318 10.1007/s10495-021-01661-5

[55] Montoya‐Zegarra JA , Russo E , Runge P , et al. AutoTube: a novel software for the automated morphometric analysis of vascular networks in tissues. Angiogenesis. 2019;22(2):223‐236. 30370470 10.1007/s10456-018-9652-3 PMC6475513

[56] Flippo KH , Lin Z , Dickey AS , et al. Deletion of a neuronal Drp1 activator protects against cerebral ischemia. J Neurosci. 2020;40(15):3119‐3129. 32144179 10.1523/JNEUROSCI.1926-19.2020 PMC7141887

[57] Branco FT , Sánchez‐Guerrero Á , Milosevic I , Raimundo N . Mitochondrial fission requires DRP1 but not dynamins. Nature. 2019;570:E34‐E42. 31217603 10.1038/s41586-019-1296-y

[58] Smirnova E , Griparic L , Shurland DL , van der Bliek AM . Dynamin‐related protein Drp1 is required for mitochondrial division in mammalian cells. Mol Biol Cell. 2001;12(8):2245‐2256. 11514614 10.1091/mbc.12.8.2245 PMC58592

[59] Flippo KH , Gnanasekaran A , Perkins GA , et al. AKAP1 protects from cerebral ischemic stroke by inhibiting Drp1‐dependent mitochondrial fission. J Neurosci. 2018;38(38):8233‐8242. 30093535 10.1523/JNEUROSCI.0649-18.2018 PMC6146498

[60] Patten DA , McGuirk S , Anilkumar U , et al. Altered mitochondrial fusion drives defensive glutathione synthesis in cells able to switch to glycolytic ATP production. Biochim Biophys Acta Mol Cell Res. 2021;1868(1):118854. 32926942 10.1016/j.bbamcr.2020.118854

[61] Sloat SR , Whitley BN , Engelhart EA , Hoppins S . Identification of a mitofusin specificity region that confers unique activities to Mfn1 and Mfn2. Mol Biol Cell. 2019;30(17):2309‐2319. 31188717 10.1091/mbc.E19-05-0291 PMC6743458

[62] Cipolat S , Martins de Brito O , Dal Zilio B , Scorrano L . OPA1 requires mitofusin 1 to promote mitochondrial fusion. Proc Natl Acad Sci USA. 2004;101(45):15927‐15932. 15509649 10.1073/pnas.0407043101 PMC528769

[63] Lee Y‐J , Jeong S‐Y , Karbowski M , Smith CL , Youle RJ . Roles of the mammalian mitochondrial fission and fusion mediators Fis1, Drp1, and Opa1 in apoptosis. Mol Biol Cell. 2004;15(11):5001‐5011. 15356267 10.1091/mbc.E04-04-0294 PMC524759

[64] Borlongan CV , Hung N , Lippert T , et al. May the force be with you: Transfer of healthy mitochondria from stem cells to stroke cells. J Cereb Blood Flow Metab. 2019;39(2):367‐370. 30375940 10.1177/0271678X18811277 PMC6365599

[65] Ramachandran R . Mitochondrial dynamics: the dynamin superfamily and execution by collusion. Semin Cell Dev Biol. 2018;76:201‐212. 28754444 10.1016/j.semcdb.2017.07.039 PMC5785577

[66] Zhao YX , Cui M , Chen SF , Dong Q , Liu XY . Amelioration of ischemic mitochondrial injury and Bax‐dependent outer membrane permeabilization by Mdivi‐1. CNS Neurosci Ther. 2014;20(6):528‐538. 24712408 10.1111/cns.12266 PMC6493009

[67] Xu Y , Wang Y , Wang G , et al. YiQiFuMai powder injection protects against ischemic stroke via inhibiting neuronal apoptosis and PKC δ /Drp1‐mediated excessive mitochondrial fission. Oxid Med Cell Longev. 2017;2017:1832093. 29435096 10.1155/2017/1832093 PMC5757147

[68] Lai Y , Lin P , Chen M , et al. Restoration of L‐OPA1 alleviates acute ischemic stroke injury in rats via inhibiting neuronal apoptosis and preserving mitochondrial function. Redox Biol. 2020;34:101503. 32199783 10.1016/j.redox.2020.101503 PMC7327985

[69] Zhang L , He Z , Zhang Q , et al. Exercise pretreatment promotes mitochondrial dynamic protein OPA1 expression after cerebral ischemia in rats. Int J Mol Sci. 2014;15(3):4453‐4463. 24633199 10.3390/ijms15034453 PMC3975407

[70] Ren K‐D , Liu W‐N , Tian J , et al. Mitochondrial E3 ubiquitin ligase 1 promotes brain injury by disturbing mitochondrial dynamics in a rat model of ischemic stroke. Eur J Pharmacol. 2019;861:172617. 31430457 10.1016/j.ejphar.2019.172617

[71] Rutkai I , Merdzo I , Wunnava SV , Curtin GT , Katakam PVG , Busija DW . Cerebrovascular function and mitochondrial bioenergetics after ischemia‐reperfusion in male rats. J Cereb Blood Flow Metab. 2019;39(6):1056‐1068. 29215305 10.1177/0271678X17745028 PMC6547195

[72] Wu X , Cui W , Guo W , et al. Acrolein aggravates secondary brain injury after intracerebral hemorrhage through Drp1‐mediated mitochondrial oxidative damage in mice. Neurosci Bull. 2020;36(10):1158‐1170. 32436179 10.1007/s12264-020-00505-7 PMC7532238

[73] Fan L‐F , He P‐Y , Peng Y‐C , et al. Mdivi‐1 ameliorates early brain injury after subarachnoid hemorrhage via the suppression of inflammation‐related blood‐brain barrier disruption and endoplasmic reticulum stress‐based apoptosis. Free Radic Biol Med. 2017;112:336‐349. 28790012 10.1016/j.freeradbiomed.2017.08.003

[74] Wu X , Luo J , Liu H , et al. Recombinant adiponectin peptide promotes neuronal survival after intracerebral haemorrhage by suppressing mitochondrial and ATF4‐CHOP apoptosis pathways in diabetic mice via Smad3 signalling inhibition. Cell Prolif. 2020;53(2):e12759. 31922310 10.1111/cpr.12759 PMC7048203

[75] Rubinsztein DC , Codogno P , Levine B . Autophagy modulation as a potential therapeutic target for diverse diseases. Nat Rev Drug Discov. 2012;11(9):709‐U784. 22935804 10.1038/nrd3802 PMC3518431

[76] Xu D , Duan H , Zhang Z , et al. The Novel Tetramethylpyrazine Bis‐nitrone (TN‐2) protects against MPTP/MPP+‐induced neurotoxicity via inhibition of mitochondrial‐dependent apoptosis. J Neuroimmune Pharmacol. 2014;9(2):245‐258. 24233519 10.1007/s11481-013-9514-0

[77] Malboosi N , Nasehi M , Hashemi M , Vaseghi S , Zarrindast M‐R . The neuroprotective effect of NeuroAid on morphine‐induced amnesia with respect to the expression of TFAM, PGC‐1α, ΔfosB and CART genes in the hippocampus of male Wistar rats. Gene. 2020;742:144601. 32198124 10.1016/j.gene.2020.144601

[78] Navazani P , Vaseghi S , Hashemi M , Shafaati M‐R , Nasehi M . Effects of treadmill exercise on the expression level of BAX, BAD, BCL‐2, BCL‐XL, TFAM, and PGC‐1 alpha in the hippocampus of thimerosal‐treated rats. Neurotox Res. 2021;39:1274‐1284. 33939098 10.1007/s12640-021-00370-w

[79] Su K‐Y , Yu CY , Chen Y‐W , et al. Rutin, a flavonoid and principal component of *Saussurea involucrata*, attenuates physical fatigue in a forced swimming mouse model. Int J Med Sci. 2014;11(5):528‐537. 24693223 10.7150/ijms.8220 PMC3970108

[80] Ranjan AK , Briyal S , Gulati A . Sovateltide (IRL‐1620) activates neuronal differentiation and prevents mitochondrial dysfunction in adult mammalian brains following stroke. Sci Rep. 2020;10(1):12721–12737. 32728189 10.1038/s41598-020-69673-w PMC7391684

[81] Chen M , Wang Z , Zhou W , et al. SIRT1/PGC‐1α signaling activation by mangiferin attenuates cerebral hypoxia/reoxygenation injury in neuroblastoma cells. Eur J Pharmacol. 2021;907:174236. 34116043 10.1016/j.ejphar.2021.174236

[82] Orgah JO , Ren J , Liu X , Orgah EA , Gao XM , Zhu Y . Danhong injection facilitates recovery of post‐stroke motion deficit via Parkin‐enhanced mitochondrial function. Restor Neurol Neurosci. 2019;37(4):375‐395. 31282440 10.3233/RNN-180828

[83] Feng J , Chen X , Lu S , et al. Naringin attenuates cerebral ischemia‐reperfusion injury through inhibiting peroxynitrite‐mediated mitophagy activation. Mol Neurobiol. 2018;55(12):9029‐9042. 29627876 10.1007/s12035-018-1027-7

[84] Zhang Y , He Y , Wu M , et al. Rehmapicroside ameliorates cerebral ischemia‐reperfusion injury via attenuating peroxynitrite‐mediated mitophagy activation. Free Radic Biol Med. 2020;160:526‐539. 32784031 10.1016/j.freeradbiomed.2020.06.034

[85] Lan R , Zhang Y , Wu T , et al. Xiao‐Xu‐Ming decoction reduced mitophagy activation and improved mitochondrial function in cerebral ischemia and reperfusion injury. Behav Neurol. 2018;2018:1‐12. 10.1155/2018/4147502 PMC6029470 30018669

[86] Wu M , Lu G , Lao Y‐Z , et al. Garciesculenxanthone B induces PINK1‐Parkin‐mediated mitophagy and prevents ischemia‐reperfusion brain injury in mice. Acta Pharmacol Sin. 2021;42(2):199‐208. 32759963 10.1038/s41401-020-0480-9 PMC8026581

[87] Tao J , Shen C , Sun Y , Chen W , Yan G . Neuroprotective effects of pinocembrin on ischemia/reperfusion‐induced brain injury by inhibiting autophagy. Biomed Pharmacother. 2018;106:1003‐1010. 30119165 10.1016/j.biopha.2018.07.026

[88] Wang L , Xiong X , Zhang X , et al. Sodium tanshinone IIA sulfonate protects against cerebral ischemia‐reperfusion injury by inhibiting autophagy and inflammation. Neuroscience. 2020;441:46‐57. 32505745 10.1016/j.neuroscience.2020.05.054

[89] Huang Q , Lou T , Wang M , et al. Compound K inhibits autophagy‐mediated apoptosis induced by oxygen and glucose deprivation/reperfusion via regulating AMPK‐mTOR pathway in neurons. Life Sci. 2020;254:117793. 32416164 10.1016/j.lfs.2020.117793

[90] Fu C , Zhang X , Lu Y , et al. Geniposide inhibits NLRP3 inflammasome activation via autophagy in BV‐2 microglial cells exposed to oxygen‐glucose deprivation/reoxygenation. Int Immunopharmacol. 2020;84:106547. 32361652 10.1016/j.intimp.2020.106547

[91] Mu F , Jiang Y , Ao F , Wu H , You Q , Chen Z . Y RapaLink‐1 plays an antithrombotic role in antiphospholipid syndrome by improving autophagy both in vivo and vitro. Biochem Biophys Res Comm. 2020;525(2):384‐391. 32093890 10.1016/j.bbrc.2020.02.084

[92] Mu Q , Zhou H , Xu Y , et al. NPD1 inhibits excessive autophagy by targeting RNF146 and wnt/beta‐catenin pathway in cerebral ischemia‐reperfusion injury. J Recept Signal Transduct. 2020;40(5):456‐463. 10.1080/10799893.2020.1756325 32326811

[93] Ma H‐X , Hou F , Chen A‐L , Li T‐T , Zhu Y‐F , Zhao Q‐P Mu‐Xiang‐You‐Fang protects PC12 cells against OGD/R‐induced autophagy via the AMPK/mTOR signaling pathway. J Ethnopharmacol. 2020;252:112583. 31978519 10.1016/j.jep.2020.112583

[94] Sun X , Wang D , Zhang T , et al. Eugenol attenuates cerebral ischemia‐reperfusion injury by enhancing autophagy via AMPK‐mTOR‐P70S6K pathway. Front Pharmacol. 2020;11:73–84. 32153404 10.3389/fphar.2020.00084 PMC7047211

[95] Zhou K , Chen J , Wu J , et al. Atractylenolide III ameliorates cerebral ischemic injury and neuroinflammation associated with inhibiting JAK2/STAT3/Drp1‐dependent mitochondrial fission in microglia. Phytomedicine. 2019;59:152922. 30981186 10.1016/j.phymed.2019.152922

[96] Li S , Sun X , Xu L , et al. Baicalin attenuates in vivo and in vitro hyperglycemia‐exacerbated ischemia/reperfusion injury by regulating mitochondrial function in a manner dependent on AMPK. Eur J Pharmacol. 2017;815:118‐126. 28743390 10.1016/j.ejphar.2017.07.041

[97] Zhou X , Wang H‐Y , Wu B , et al. Ginkgolide K attenuates neuronal injury after ischemic stroke by inhibiting mitochondrial fission and GSK‐3 beta‐dependent increases in mitochondrial membrane permeability. Oncotarget. 2017;8(27):44682‐44693. 28591721 10.18632/oncotarget.17967 PMC5546510

[98] Chen S , Sun M , Zhao X , et al. Neuroprotection of hydroxysafflor yellow A in experimental cerebral ischemia/reperfusion injury via metabolic inhibition of phenylalanine and mitochondrial biogenesis. Mol Med Rep. 2019;19(4):3009‐3020. 30816517 10.3892/mmr.2019.9959 PMC6423596

[99] Xu B , Zhu L , Chu J , et al. Esculetin improves cognitive impairments induced by transient cerebral ischaemia and reperfusion in mice via regulation of mitochondrial fragmentation and mitophagy. Behav Brain Res. 2019;372:112007. 31238056 10.1016/j.bbr.2019.112007

[100] Liu W‐J , Jiang H‐F , Ul Rehman F , et al. Lycium barbarum polysaccharides decrease hyperglycemia‐aggravated ischemic brain injury through maintaining mitochondrial fission and fusion balance. Int J Biol Sci. 2017;13(7):901‐910. 28808422 10.7150/ijbs.18404 PMC5555107

[101] Li JM , Li Y , Ogle M , et al. DL‐3‐n‐butylphthalide prevents neuronal cell death after focal cerebral ischemia in mice via the JNK pathway. Brain Res. 2010;1359:216‐226. 20800583 10.1016/j.brainres.2010.08.061 PMC3099257

[102] Suomalainen A , Battersby BJ . Mitochondrial diseases: the contribution of organelle stress responses to pathology. Nat Rev Mol Cell Biol. 2018;19(2):77‐92. 28792006 10.1038/nrm.2017.66

[103] Tucker D , Lu Y , Zhang Q . From mitochondrial function to neuroprotection‐an emerging role for methylene blue. Mol Neurobiol. 2018;55(6):5137‐5153. 28840449 10.1007/s12035-017-0712-2 PMC5826781

[104] Herzig S , Shaw RJ . AMPK: guardian of metabolism and mitochondrial homeostasis. Nat Rev Mol Cell Biol. 2018;19(2):121‐135. 28974774 10.1038/nrm.2017.95 PMC5780224

